# Intestinal Microbiota-Derived GABA Mediates Interleukin-17 Expression during Enterotoxigenic *Escherichia coli* Infection

**DOI:** 10.3389/fimmu.2016.00685

**Published:** 2017-01-16

**Authors:** Wenkai Ren, Jie Yin, Hao Xiao, Shuai Chen, Gang Liu, Bie Tan, Nengzhang Li, Yuanyi Peng, Tiejun Li, Benhua Zeng, Wenxia Li, Hong Wei, Zhinan Yin, Guoyao Wu, Philip R. Hardwidge, Yulong Yin

**Affiliations:** ^1^National Engineering Laboratory for Pollution Control and Waste Utilization in Livestock and Poultry Production, Institute of Subtropical Agriculture, The Chinese Academy of Sciences, Changsha, China; ^2^Key Laboratory of Agro-Ecology, Institute of Subtropical Agriculture, The Chinese Academy of Sciences, Changsha, China; ^3^University of the Chinese Academy of Sciences, Beijing, China; ^4^Chongqing Key Laboratory of Forage and Herbivorce, College of Animal Science and Technology, Southwest University, Chongqing, China; ^5^Department of Laboratory Animal Science, College of Basic Medicine Science, Third Military Medical University, Chongqing, China; ^6^Biomedical Translational Research Institute, Jinan University, Guangzhou, China; ^7^Department of Animal Science, Texas A&M University, College Station, TX, USA; ^8^Department of Diagnostic Medicine/Pathobiology, Kansas State University, Manhattan, KS, USA; ^9^College of Animal Science, South China Agricultural University, Guangzhou, China

**Keywords:** intestinal microbiota, GABA, IL-17, mTORC1, ETEC, *Lactococcus lactis*

## Abstract

Intestinal microbiota has critical importance in pathogenesis of intestinal infection; however, the role of intestinal microbiota in intestinal immunity during enterotoxigenic *Escherichia coli* (ETEC) infection is poorly understood. The present study tested the hypothesis that the intestinal microbiota is associated with intestinal interleukin-17 (IL-17) expression in response to ETEC infection. Here, we found ETEC infection induced expression of intestinal IL-17 and dysbiosis of intestinal microbiota, increasing abundance of γ-aminobutyric acid (GABA)-producing *Lactococcus lactis* subsp. *lactis*. Antibiotics treatment in mice lowered the expression of intestinal IL-17 during ETEC infection, while GABA or *L. lactis* subsp. *lactis* administration restored the expression of intestinal IL-17. *L. lactis* subsp. *lactis* administration also promoted expression of intestinal IL-17 in germ-free mice during ETEC infection. GABA enhanced intestinal IL-17 expression in the context of ETEC infection through activating mechanistic target of rapamycin complex 1 (mTORC1)-ribosomal protein S6 kinase 1 (S6K1) signaling. GABA–mTORC1 signaling also affected intestinal IL-17 expression in response to *Citrobacter rodentium* infection and in drug-induced model of intestinal inflammation. These findings highlight the importance of intestinal GABA signaling in intestinal IL-17 expression during intestinal infection and indicate the potential of intestinal microbiota-GABA signaling in IL-17-associated intestinal diseases.

## Introduction

The intestinal microbiota has critical importance in intestinal infections by increasing colonization resistance and promoting pathogen clearance after infection (1, 2). For example, *Clostridium difficile* infection, which is the leading health care-associated illness, usually follows the disruption of the indigenous gut microbiota after antibiotic treatment, leading to the loss of colonization resistance against *C. difficile* (2–4). The gut microbiota also affects intestinal infections by mediating the host innate and adaptive immune responses (5, 6). For example, germ-free mice are highly susceptible to *Listeria monocytogenes* infection because of impaired activation and accumulation of phagocytes to the site of infection (6).

Interleukin (IL)-17 promotes local chemokine production to recruit monocytes and neutrophils to sites of inflammation and is thus important in mediating protection against pathogens, especially against extracellular pathogens (7). IL-17 combats the microbes attacking epithelial layers and has critical functions in protecting against bacterial infection at mucosal sites (8). IL-17 is also thought to play major roles in the development and pathogenesis of various autoimmune diseases, including rheumatoid arthritis, psoriasis vulgaris, multiple sclerosis, and inflammatory bowel disease (9, 10).

Intestinal expression of IL-17 is induced after intestinal infection by most pathogens (11–13). For example, *Citrobacter rodentium* or *Salmonella* infection promotes intestinal IL-17 expression by enteric innate T helper type 17 (iTh17) cells (12). Enterotoxigenic *Escherichia coli* (ETEC) is a common cause of diarrhea in humans and livestock (14). Previous investigations have found that ETEC infection triggers intestinal IL-17 expression (15, 16). However, the underlying mechanisms are largely unknown. The present study tested the hypothesis that the intestinal microbiota is associated with intestinal IL-17 expression in response to ETEC infection.

We confirmed that ETEC promotes intestinal IL-17 expression in piglets and mice and showed that the activation of the mechanistic target of rapamycin complex 1 (mTORC1)-growth factor independence 1 (GFI-1) signaling mediates intestinal IL-17 expression in the context of ETEC infection. We clarified that γ-aminobutyric acid (GABA) signaling is critical to activating the mTORC1–GFI-1–IL-17 pathway during ETEC infection, and this signaling is largely dependent on the intestinal GABA-producing strain *Lactococcus lactis* subsp. *lactis*.

## Materials and Methods

### Bacterial Strains and Antibodies

This study involved the use of an *E. coli* F4-producing strain W25K (hereafter referred as ETEC; O149:K91, K88ac; LT, STb, EAST), which was isolated from a piglet with diarrhea (17). ETEC W470 (O4:F18; STa, STb, LT), W817 (O107:F18; STb), and W616 (F18; STa) were also isolated from piglets with diarrhea, while the Shiga-like toxin producing *E. coli* (W197, SLT-IIe) was isolated from a piglet with edema disease (18). These strains of bacteria were cultured in LB medium. *L. lactis* subsp. *lactis* (ATCC19435) was cultured in M17 medium. *C. rodentium* (DBS100) was cultured in LB medium. Antibodies against RAR-related orphan receptor gamma t (RORγt) (Sc-14196), forkhead box P3 (Foxp3) (Sc-28705), growth factor independent 1 (GFI-1) (Sc-8558), early growth response protein 2 (EGR-2) (Sc-20690), p85 (Sc-1637), phosphorylated protein kinase B (Akt) (Sc7985-R), GAT-2 (Sc-7668), actin (Sc-47778), and proliferating cell nuclear antigen (PCNA) (Sc-56) were purchased from Santa Cruz Biotechnology, Inc. (Dallas, TX, USA). Antibodies against mTOR (CST 2972), p-mTOR (CST 5536), p-p70 S6 Kinase (CST 9205), p-4E-BP1 (CST 9451), p-AMP-activated protein kinase (AMPK) (CST 2535), hypoxia-inducible factor 1 α (HIF-1α) (CST 14179), and p70 S6 Kinase 2 (CST 14130) were purchased from Cell Signaling Technology (Danvers, MA, USA).

### ETEC Infection in Piglets

This study was approved and conducted according to the guidelines of the Institute of Subtropical Agriculture, Chinese Academy of Sciences and Southwest University. Piglets (Landrace Yorkshire; 18 days old) were purchased from ZhengDa Co., Chongqing, China. ETEC infection in piglets was established according to previous reports (19, 20). The jejunum samples were collected. Samples were stored at −80°C until processing.

### Mice

TCR delta knockout mice were provided by Prof. Zhinan Yin, from Jinan University (Guangzhou, China). Rag 1 knockout mice were bought from Nanjing University (Nanjing, China). Germ-free mice were generated and provided by Prof. Hong Wei, from Third Military Medical University (Chongqing, China), and these mice were maintained in sterile Trexler-type isolators. ICR mice (6 weeks of age) were purchased from SLAC Laboratory Animal Central (Changsha, China). Experiments in mice were conducted according to the guidelines of the Laboratory Animal Ethical Commission of the Chinese Academy of Sciences.

### ETEC Infection in Mice

Mice were orally gavaged with 10^8^ CFUs of ETEC or other strains of *E. coli*. At 6-h postinfection, mice were sacrificed to collect the jejunum, ileum, and mesenteric lymph node. In some experiments, mice were treated with rapamycin (Fisher Scientific) at 2.5 mg/kg/d (i.p. injection) for six consecutive days prior to other manipulations. In experiments involving the inhibition of GABA signaling, mice were intraperitoneally injected with 20 mg/kg bicuculline (Dalian Meilun Bio. Tech. Co., Ltd., Dalian, China), 120 mg/kg CGP-35348 (Tocris Bioscience), 80 mg/kg l-allylglycine (Aladdin Industrial Corporation, China), or 100 mg/kg semicarbazide (Sangon Biotech Co., Ltd., Shanghai, China) at 30 min prior to other manipulations. For the inhibition of S6K1, mice were intraperitoneally injected with 75 mg/kg/d PF-4708671 (S2163, Selleck) for six consecutive days. In some experiments, mice were intraperitoneally injected with GABA (Aladdin Industrial Corporation, China) at dosages of 40 μg//kg to 40 mg/kg at 30 min prior to other manipulations [the physiologic level of GABA in mouse serum is about 2 mg/kg (21), while in human (adults), it is about 100–400 µg/kg]. For antibiotics treatment, mice received drinking water containing streptomycin (1.0 g/L, Sigma), ampicillin (1.0 g/L, Sigma), gentamicin (1.0 g/L, Sigma), and vancomycin (0.5 g/L, Sigma) for 1 week before ETEC infection. For the supplementation of GABA to antibiotics-treated mice, mice received drinking water containing antibiotics (1.0 g/L streptomycin, 1.0 g/L ampicillin, 1.0 g/L gentamicin, and 0.5 g/L vancomycin) and GABA (1.0 g/L or 5.0 g/L) for 1 week before ETEC infection. For *L. lactis* subsp. *lactis* inoculation into antibiotics-treated mice, mice received drinking water containing antibiotics (1.0 g/L streptomycin, 1.0 g/L ampicillin, 1.0 g/L gentamicin, and 0.5 g/L vancomycin) for 6 days, and then orally inoculated with *L. lactis* subsp. *lactis* at a dosage of 10^8^ CFUs at 24 h before ETEC infection. For *L. lactis* subsp. *lactis* inoculation into germ-free mice, mice were orally inoculated with *L. lactis* subsp. *lactis* at a dosage of 10^8^ CFUs at 7 days before ETEC infection.

### *L. lactis* Subsp. *lactis* Inoculation in Mice

For *L. lactis* subsp. *lactis* inoculation into normal mice, mice were orally inoculated with *L. lactis* subsp. *lactis* at a dosage of 10^8^ CFUs for five consecutive days. For GABA signaling inhibition, mice were intraperitoneally injected with 120 mg/kg CGP-35348 at 5 days postinoculation. The jejunum samples were collected for further analysis at 5 days postinoculation (6-h post-CGP-35348 treatment).

### *L. lactis* Subsp. *lactis* Inoculation in Piglets

For *L. lactis* subsp. *lactis* inoculation in piglets, piglets were orally inoculated with *L. lactis* subsp. *lactis* at dose of 10^10^, while the control piglets received some volume of M17 medium. The jejunum samples were collected for analysis at 7 days postinoculation.

### *C. rodentium* Infection in Mice

Mice (6 weeks of age) were orally gavaged with 10^8^ CFUs of *C. rodentium* (DBS100). For rapamycin treatment, mice were treated with rapamycin at 2.5 mg/kg/d (i.p. injection) at 1–7 days postinfection. For semicarbazide treatment, mice were treated with semicarbazide at 100 mg/kg/d (i.p. injection) at 1, 3, 5, and 7 days postinfection. In some experiments, mice were intraperitoneally injected with 20 mg/kg bicuculline or 120 mg/kg CGP-35348 at 7 days postinfection. The colon samples from all groups were collected for further analysis at 7 days postinfection (6-h post-bicuculline or CGP-35348 treatment).

### 5-Fluorouracil Treatment in Mice

ICR mice (6 weeks of age) were intraperitoneally injected with 300 mg/kg 5-fluorouracil (Sigma-Aldrich). For the inhibition of GABA signaling, mice received intraperitoneal administration of 20 mg/kg bicuculline or 100 mg/kg semicarbazide at 30 min prior to 5-fluorouracil treatment. The jejunum was collected at 6-h posttreatment for further analysis.

### Lymphocyte Isolation from Jejunum

Lamina propria lymphocytes from jejunum were obtained according to previous procedures (12, 22, 23). Briefly, jejunum tissue was extracted, opened longitudinally to wash luminal contents. Jejunum (cleared the visible PP) was cut into 0.5- to 1-cm segments that were incubated three times (37°C, 20 min, 250 rpm) in HBSS/HEPES medium (Life Technologies) containing 5 mM EDTA to remove the epithelial cells. Thereafter, the tissue segments were minced and digested in digestion buffer [HBSS + 5% FBS containing type VIII collagenase at 1.5 mg/mL (Sigma)] for 30-min incubations at 37°C. Digested material was passed through a 100-µm cell strainer, and the cells were collected by centrifugation, washed twice in DMEM, and then passed through a 40-µm cell strainer to obtain lamina propria lymphocytes from the jejunum.

### Flow Cytometry Analysis

Lymphocytes isolated from mouse jejunal lamina propria were stained with cell surface markers of CD3 (FITC-CD3, 100203, Biolegend), CD4 (PE-CD4, 100407, Biolegend), or TCR γδ (PE-TCR γδ, 118107, Biolegend). For the analysis of intracellular cytokine, lymphocytes or CD4^+^ T cells were stimulated with PMA (50 ng/mL), ionomycin (1 µg/mL), and monensin (3 µg/mL) for 5 h. After staining with cell surface markers, intracellular cytokine staining was performed with a fixation and permeabilization kit (eBioscience) and IL-17A Ab (APC-IL-17A, 506195, Biolegend) in accordance with the manufacturer’s instructions. Flow cytometry was performed on a FACSCalibur (BD Biosciences) and data were analyzed using the FlowJo Software (Tree Star).

### Metabolite Profiling Analysis

Metabolite concentrations in piglet jejunal samples were quantified using gas chromatography/mass spectrometry (GC–MS) according to the previous work (19).

### RT-PCR

Real-time PCR was performed according to our previous study (20, 24). Primers (Table S1 in Supplementary Material) were selected according to previous references. β-Actin was used as an internal control to normalize target gene transcript levels.

### Immunoblotting

Immunoblotting was performed according to our previous study (20, 24). Signal intensity was digitally quantified and normalized to actin or PCNA protein abundance.

### Th17 Cell Differentiation

Naive CD4^+^ T cells were isolated from mouse splenocytes using a CD4^+^CD62L^+^ T cell isolation kit II (Miltenyi Biotec, purity > 95%). For Th17 differentiation, cells were stimulated with anti-CD3 (2 µg/mL, 100313, Biolegend) and anti-CD28 (2.0 µg/mL, 102111, Biolegend) supplemented with 5 ng/ml TGF-β1 (7666-MB-005, RD), 20 ng/mL IL-6 (575702, Biolegend), 10 µg/mL anti-IFN-γ (505812, Biolegend), and 10 µg/mL anti-IL-4 (504107, Biolegend) for 1–3 days. l-Allylglycine, semicarbazide, and rapamycin were used at 5 mM, 4 mM, and 3 µM, respectively.

### Statistical Analyses

Data shown are the means ± SEM. Data between two groups were analyzed by unpaired *t* test (Prism 6.0) if the data were in Gaussian distribution and had equal variance, or by unpaired *t* test with Welch’s correction (Prism 6.0) if the data were in Gaussian distribution but with unequal variance, or by non-parametric test (Mann–Whitney *U* test, Prism 6.0) if the data were not normally distributed. Data among more than two groups were analyzed by the one-way ANOVA followed by Dunnett multiple comparisons (Prism 6.0) if the data were in Gaussian distribution and had equal variance, or analyzed by Kruskal–Wallis followed by Dunn’s multiple comparisons (Prism 6.0) if the data were not normally distributed. The Gaussian distribution of data was analyzed by D’Agostino–Pearson omnibus normality test (Prism 6.0) and Kolmogorov–Smirnov test (Prism 6.0). The variance of data was analyzed by homogeneity of variance test (SPSS 22.0) or Brown–Forsythe test (Prism 6.0). The Bonferroni correction was applied for multiple pairwise comparisons. Differences with *p* < 0.05 were considered significant.

## Results

### ETEC Increases IL-17 Expression in Piglets and Mice

**Figure 1:**
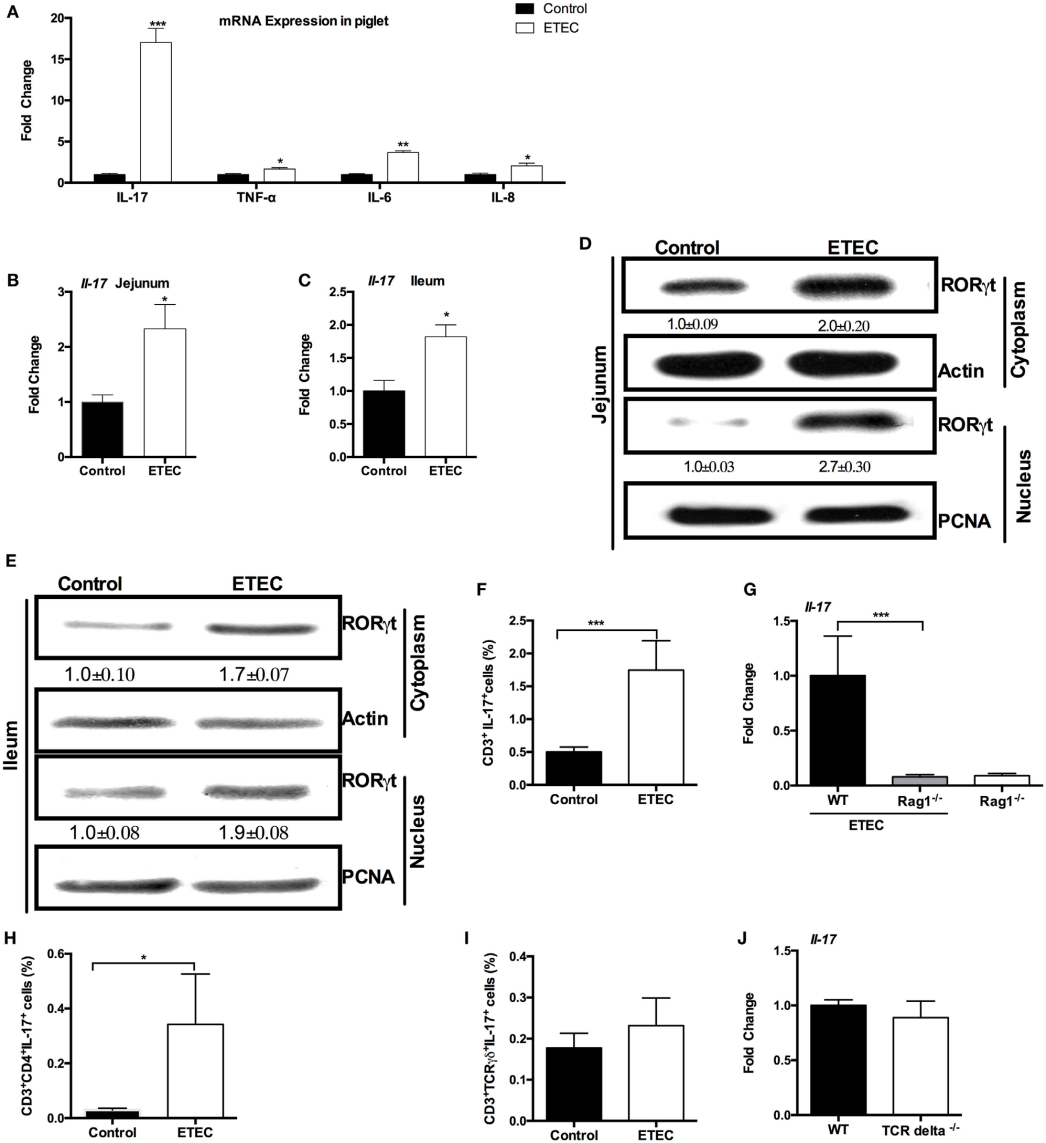
**Enterotoxigenic *Escherichia coli* (ETEC) induces intestinal interleukin-17 (IL-17) expression**. **(A)** Relative mRNA expression of indicated cytokines in piglet jejunum samples (*n* = 6). **(B)** Relative mRNA expression of IL-17 in mouse jejunum. **(C)** Relative mRNA expression of IL-17 in mouse ileum. **(D)** Nuclear and cytoplasmic abundance of RAR-related orphan receptor gamma t (RORγt) in mouse jejunum samples. In this, and in all subsequent figures, the statistical data (mean ± SEM; *n* = 5) below the image indicate the relative band amount of indicated protein obtained by dividing the actin or proliferating cell nuclear antigen (PCNA) band intensity by the indicated band intensity in each lane. **(E)** Nuclear and cytoplasmic abundance of RORγt in mouse ileum samples. **(F,H,I)** ICR mice were infected with ETEC for 6 h, and lymphocytes from the jejunal lamina propria were isolated and stimulated for 5 h with PMA, ionomycin, and monensin, followed by flow cytometry analysis of the frequency of CD3^+^IL-17^+^ cells **(F)**, CD3^+^CD4^+^IL-17^+^ cells **(H)**, and CD3^+^TCR γδ^+^IL-17^+^ cells **(I)**. **(G)** Relative mRNA expression of IL-17 in Rag1^−/−^ mouse jejunum at 6-h post-ETEC infection. **(J)** Relative mRNA expression of IL-17 in TCR delta^−/−^ mouse jejunum at 6-h post-ETEC infection. *indicates a statistically significant difference between two groups (*p* < 0.05), while **indicates *p* < 0.01, and ***indicates *p* < 0.001 [unpaired *t* test **(A,J)**; Mann–Whitney test **(F,H,I)**; Kruskal–Wallis test **(G)**; Bonferroni correction was also used for **(A)**]. Data shown are representative of three independent experiments with *n* = 10 **(B,C)** or 5 **(D,E)** in each experiment, or representatives of two independent experiments with *n* = 4–7 **(F–J)** in each experiment.

Enterotoxigenic *Escherichia coli* infection is known to promote IL-17 expression in piglets (15, 16), a finding we also validated. We infected Landrace × Yorkshire piglets with ETEC W25K, a strain originally isolated from a piglet with diarrhea (17). IL-17 mRNA expression in the porcine jejunum was higher (about 17-fold) in piglets infected with ETEC, as compared with uninfected controls (Figure 1A). Increased mRNA expression of other proinflammatory cytokines, including IL-6, IL-8, and TNF-α, was also detected in the jejunum after ETEC infection (Figure 1A).

In mice, at 6-h postinfection, ETEC colonized in the jejunum with load of about 5.9 log_10_ CFU/g and promoted the expression of *Il-17* in the jejunum (Figure 1B). ETEC also promoted the expression of *Il-17* in the ileum (Figure 1C). ETEC infection enhanced the abundance of RORγt (a key transcriptional regulator of Th17 cells) in the cytoplasm and nuclei of mouse jejunum (Figure 1D) and ileum (Figure 1E).

Although the best-characterized cellular source of IL-17 is Th17 cells, a number of adaptive and innate immune cells, such as γδ T cells, natural killer T (NKT) cells, and even Paneth cells, are also the sources of IL-17 (11, 13). Similar to previous conclusion that the increased IL-17^+^ cells at 5-h post-*E. coli* infection are CD3^+^ cells (25), we found that most of IL-17^+^ cells from ETEC-infected mouse jejunum were CD3^+^ cells. Thus, the ETEC-infected mice had a higher percentage of CD3^+^IL-17^+^ cells in the jejunum compared with the controls (Figure 1F), suggesting that the increased expression of IL-17 at 6-h post-ETEC infection comes from T cells. Indeed, ETEC infection had little effect on the expression of *Il-17* in the jejunum in Rag 1 knockout mice (mature T cell-depleted mice), while wild-type mice had higher expression of *Il-17* in the jejunum compared to Rag 1 knockout mice during ETEC infection (Figure 1G). Unlike the previous study, which shows that the increased IL-17 is mainly produced by γδ T cells during early *E. coli* infection (25), we observed a higher percentage of CD3^+^CD4^+^IL-17^+^ cells in the jejunum from ETEC-infected mice as compared with uninfected mice, while the percentage of CD3^+^TCR γδ^+^IL-17^+^ cells in the jejunum did not differ between ETEC-infected mice and control mice (Figures 1H,I). Notably, *Il-17* expression in the jejunum after 6 h of ETEC infection was similar between TCR delta^−/−^ mice (γδ T cell-depleted mice) and wild-type mice (Figure 1J). In conclusion, ETEC infection promotes the intestinal mRNA expression of IL-17 in piglets and mice.

### ETEC Promotes IL-17 Expression through mTORC1 Activation

**Figure 2:**
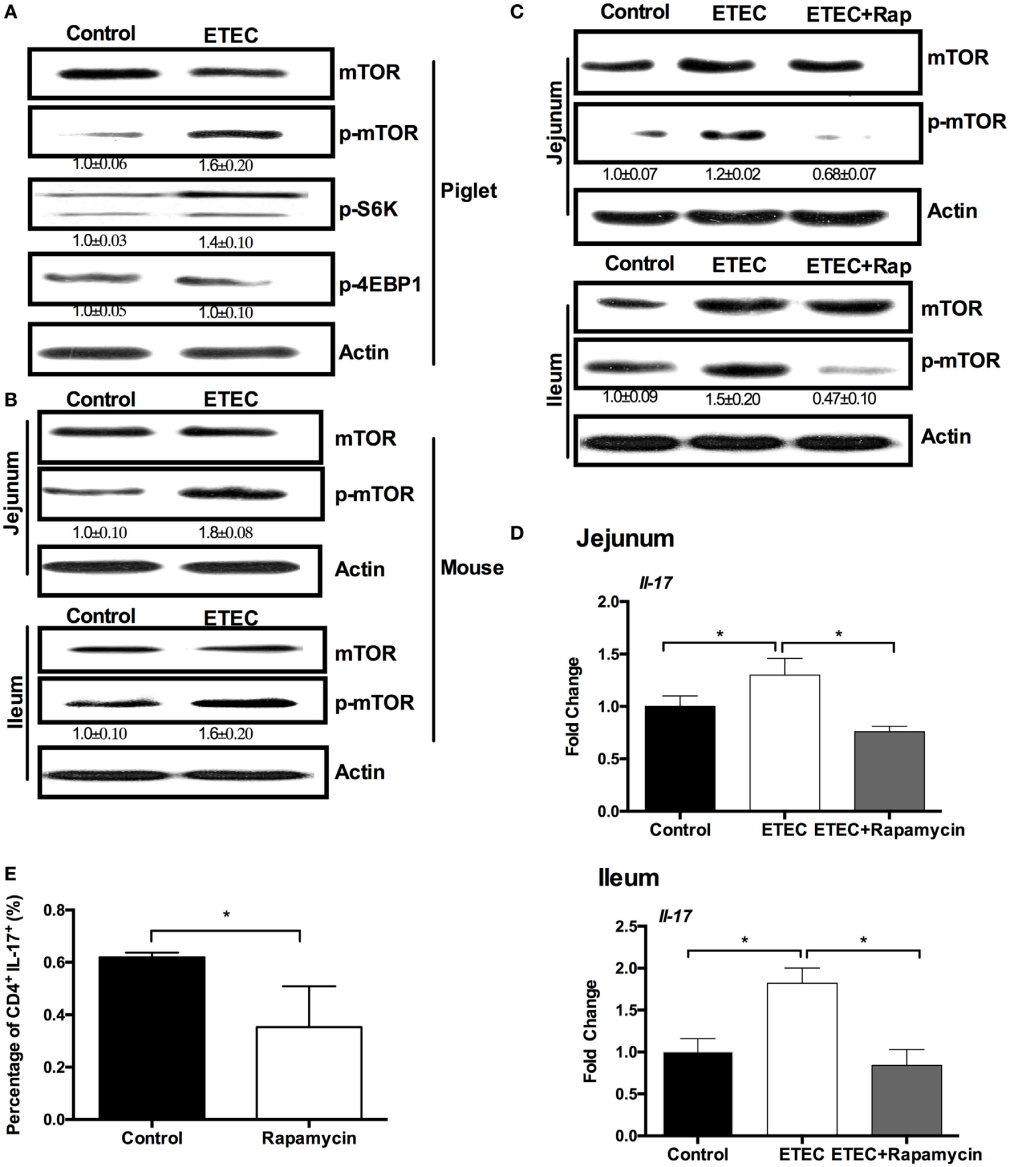
**Enterotoxigenic *Escherichia coli* (ETEC) promotes intestinal interleukin-17 (IL-17) mRNA expression through mechanistic target of rapamycin complex 1 (mTORC1) activation**. **(A)** The activation of mTOR pathway in piglet jejunum (*n* = 5). **(B)** The activation of mTOR pathways in mouse jejunum or ileum. **(C)** Rapamycin pretreatment inhibits mTOR activation in mouse jejunum and ileum. **(D)** mRNA expression of IL-17 in mouse jejunum and ileum after rapamycin treatment. **(E)** Intracellular staining of the expression of IL-17 by CD4^+^ T cells cultured under the Th17-inducing conditions with or without rapamycin for 24 h. *indicates a statistically significant difference between two groups (*p* < 0.05) [unpaired *t* test **(A,B)**; one-way ANOVA **(C,D)**; Mann–Whitney test **(E)**]. Data shown are representative of four independent experiments with *n* = 5 **(B,C)** or 10 **(D)** in each experiment, or representatives of three independent experiments with *n* = 3 **(E)** in each experiment.

A previous study has shown that ETEC infection inhibits the activation of NF-κB and mitogen-activated protein kinases pathways in the jejunum (20), indicating that new pathways are associated with expression of IL-17 during ETEC infection. Previous proteomic analysis indicated that ETEC infection upregulates ribosomal protein S6 kinase in the piglet jejunum (20), which suggests that ETEC infection may activate mTORC1 signaling. The mTORC1 has a critical role in Th17 responses and in IL-17 expression (26). Indeed, the mTORC1 pathway was activated in piglets infected with ETEC, based on the higher abundance of phosphorylated mTORC1 and its downstream target S6K (Figure 2A). Phosphorylated mTORC1 abundance was also higher in ETEC-infected mice than that in uninfected controls (Figure 2B). To validate the possible roles of mTORC1 signaling in the expression of IL-17 during ETEC infection, this study used rapamycin to inhibit the activation of mTORC1. Rapamycin treatment prior to ETEC infection prevented the increase in mTORC1 phosphorylation in the jejunum and ileum (Figure 2C) and reversed the increased *Il-17* expression in mice infected with ETEC (Figure 2D). Similar to a previous report (27), rapamycin also inhibited Th17 cell differentiation from naïve T cells under Th17 polarization conditions *in vitro* (Figures 2E). In conclusion, ETEC infection promotes IL-17 expression through the activation of mTORC1 pathway.

### ETEC Promotes IL-17 Expression through the mTORC1–EGR-2 Pathway

**Figure 3:**
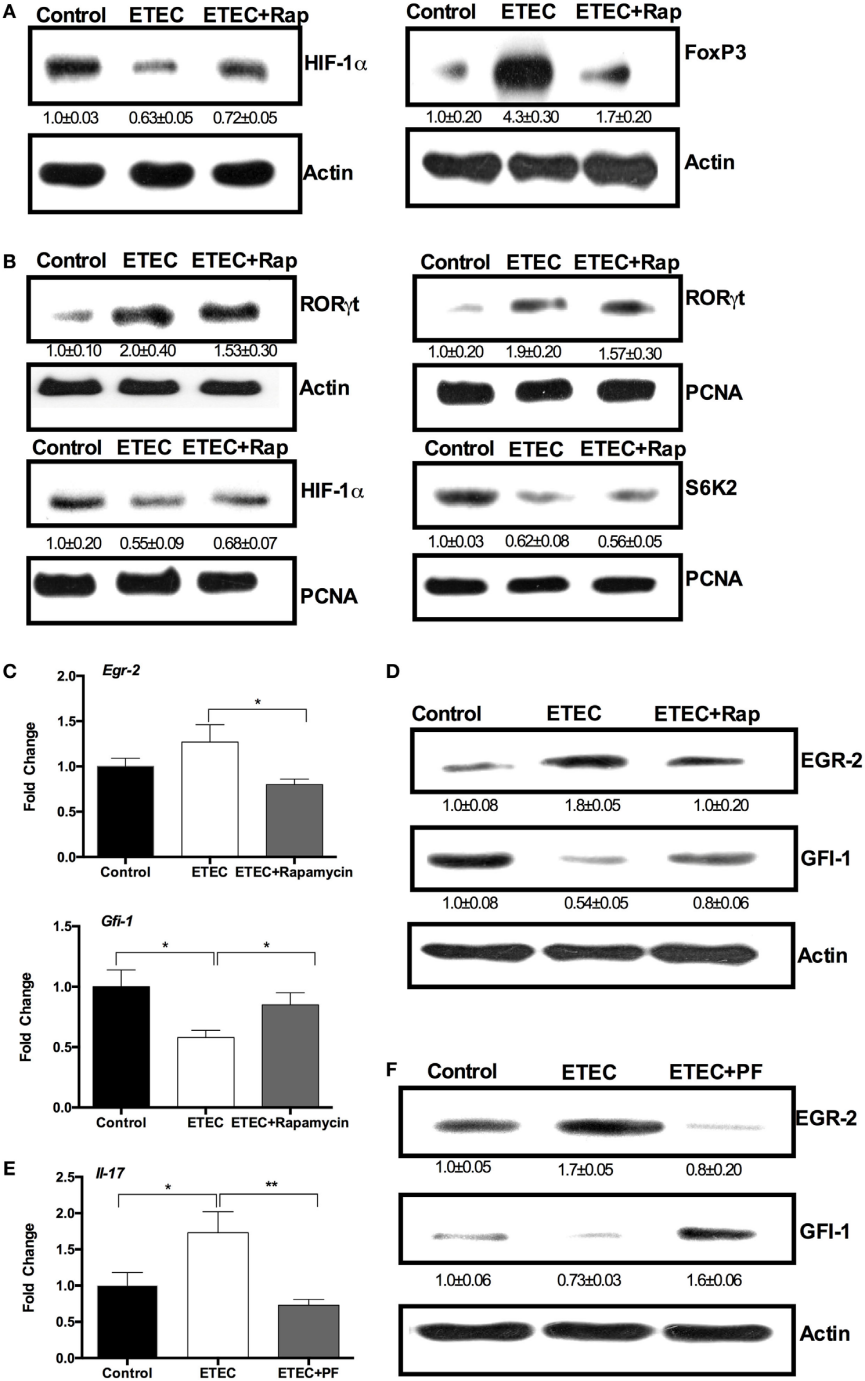
**Enterotoxigenic *Escherichia coli* (ETEC) promotes interleukin-17 (IL-17) expression through the mechanistic target of rapamycin complex 1 (mTORC1)–early growth response protein 2 (EGR-2) pathway**. **(A)** Hypoxia-inducible factor 1 α (HIF-1α) and Foxp3 protein abundance (*n* = 5). **(B)** RORγt, HIF-1α, and S6K2 abundance (*n* = 5). **(C)** EGR-2 and GFI-1 mRNA expression (*n* = 10). **(D)** EGR-2 and GFI-1 protein abundance (*n* = 5). **(E)** IL-17 mRNA expression in mouse jejunum (*n* = 10). **(F)** EGR-2 and GFI-1 abundance (*n* = 5). All these indicators were analyzed by RT-PCR (mRNA level) and immunblotting (protein level) in mouse jejunum at 6 h of post-ETEC infection. *indicates a statistically significant difference between two groups (*p* < 0.05), while **indicates *p* < 0.01 [one-way ANOVA **(A–F)**].

Among the downstream targets of mTORC1 signaling, HIF-1α and S6K (S6K1 and S6K2) can regulate IL-17 expression (26). mTORC1 signaling activates HIF-1α, which promotes IL-17 expression by activating RORγt and mediating Foxp3 degradation (26). ETEC infection decreased HIF-1α abundance and increased Foxp3 abundance in the cytoplasm of mouse jejunum compared to the controls (Figure 3A). Rapamycin treatment before ETEC infection increased HIF-1α abundance and lowered Foxp3 abundance (Figure 3A). S6K2 (the nuclear-localized counterpart of S6K1) binds to RORγt to promote the nuclear translocation of RORγt, which can complex with HIF-1α and p300 in the nucleus to promote IL-17 expression (26). Although ETEC infection increased RORγt abundance, rapamycin had no effect on RORγt abundance (Figure 3B, *top*). Similarly, rapamycin did not affect on the abundance of HIF-1α and S6K2 in the nucleus of the mouse jejunum (Figure 3B, *bottom*), although ETEC infection reduced the nuclear abundance of HIF-1α and S6K2 in mouse jejunum (Figure 3B, *bottom*).

S6K1 promotes the expression of EGR-2, resulting in the inhibition of the GFI-1, which negatively regulates IL-17 expression without affecting *Rorc* expression (26). Although ETEC infection had little affect on *Egr-2* expression in the mouse jejunum, rapamycin reduced *Egr-2* expression in the mouse jejunum (Figure 3C). ETEC infection reduced *Gfi-1* expression in the mouse jejunum, while rapamycin prevented this reduction (Figure 3C). At the protein level, ETEC infection increased the abundance of EGR-2 and lowered the abundance of GFI-1 in the mouse jejunum, while rapamycin abolished the effects of ETEC infection (Figure 3D). These results indicate that ETEC induces intestinal IL-17 expression may through the S6K1–EGR-2–GFI-1 axis. Intriguingly, ETEC infection increased *Il-17* expression in the jejunum, while PF-4708671 (S6K1 specific inhibitor) reduced *Il-17* expression in the jejunum in ETEC-infected mice (Figure 3E). ETEC infection increased the abundance of EGR-2 and decreased the abundance of GFI-1 in the mouse jejunum, while PF-4708671 treatment reversed this change during ETEC infection in mouse jejunum (Figure 3F). These data indicate ETEC promotes the IL-17 expression in the jejunum through the mTOR–S6K1–GFI-1 axis.

### ETEC Promotes IL-17 Expression through GABA–mTORC1–EGR-2 Signaling

**Figure 4:**
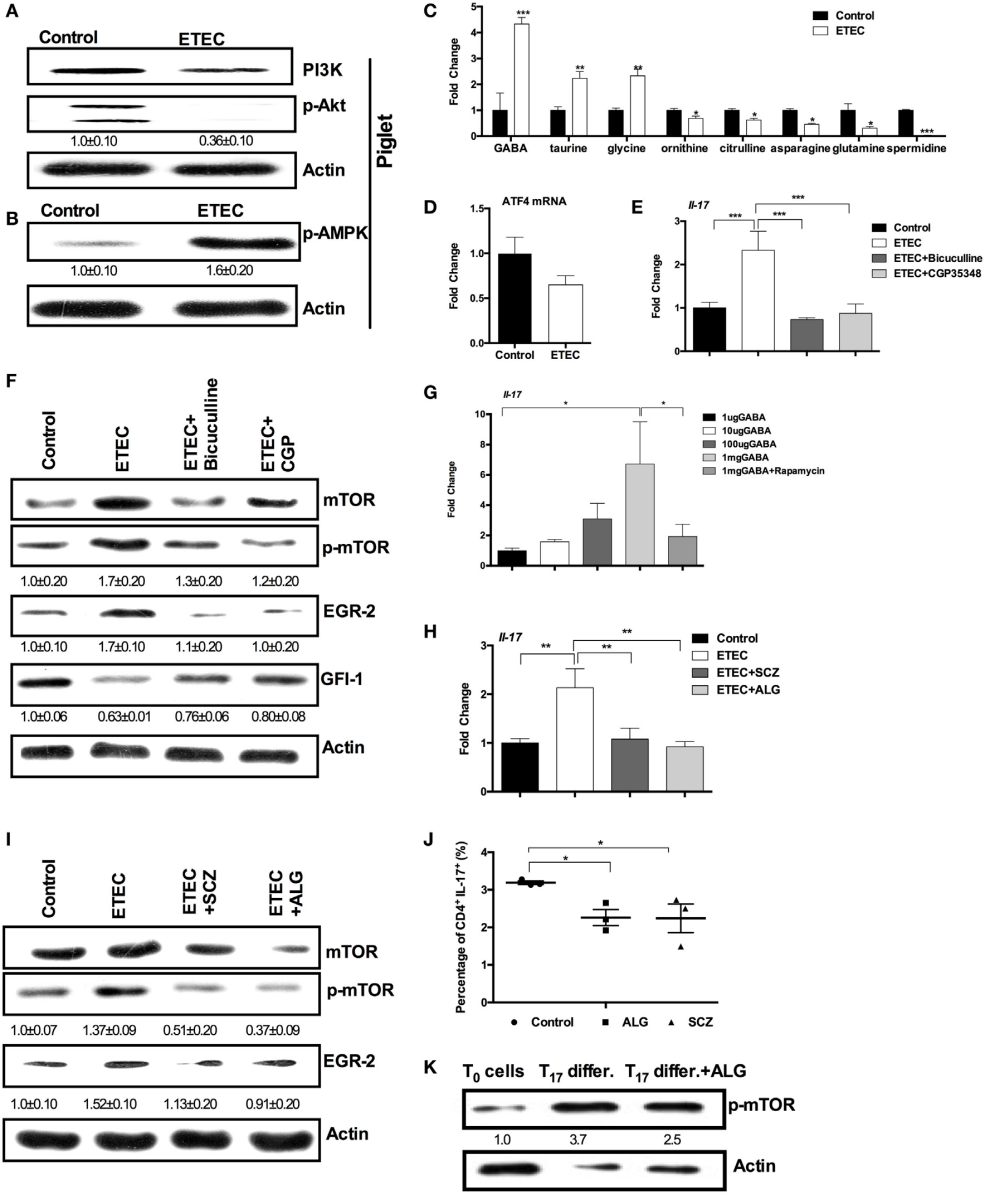
**Enterotoxigenic *Escherichia coli* (ETEC) promotes interleukin-17 (IL-17) expression through γ-aminobutyric acid (GABA)-mechanistic target of rapamycin complex 1 (mTORC1) signaling**. **(A)** The activation of PI3K-p-Akt pathway in piglet jejunum (*n* = 5). **(B)** The abundance of phosphorylated AMP-activated protein kinase (AMPK) in piglet jejunum (*n* = 5). **(C)** Relative amino acid concentrations in diarrheal piglets compared with uninfected piglets (*n* = 6). **(D)** Activating transcription factor 4 (ATF4) mRNA expression in piglet jejunum (*n* = 6). **(E)** mRNA expression of IL-17 after bicuculline or CGP-35348 treatment (*n* = 10). **(F)** mTORC1–GFI-1 activation after bicuculline or CGP-35348 treatment (*n* = 5). **(G)** mRNA expression of IL-17 after GABA injection (*n* = 10). **(H)** mRNA expression of IL-17 after l-allylglycine or semicarbazide treatment (*n* = 10). **(I)** mTORC1–early growth response protein 2 (EGR-2) activation after l-allylglycine or semicarbazide treatment (*n* = 5). **(J)** Intracellular staining of the expression of IL-17 by CD4^+^ T cells cultured under the Th17-inducing conditions with or without allyglycine or semicarbazide for 3 days. Results shown are representative of three independent experiments with *n* = 3 in each experiment. **(K)** The abundance of phosphorylated mTOR. Naïve CD4^+^ T cells were isolated (T_0_ cells) and cultured under the Th17-inducing conditions without (Th17 differ.) or with allyglycine (Th17 differ. + ALG) for 3 days. Results shown are representative of three independent experiments. The numbers indicate the relative band amount of p-mTOR protein in each sample obtained by dividing the actin band intensity by the p-mTOR band intensity in each lane. *indicates a statistically significant difference between two groups (*p* < 0.05), while **indicates *p* < 0.01, and ***indicates *p* < 0.001 [unpaired *t* test **(A–D)**; one-way ANOVA **(E–I)**; Kruskal–Wallis test **(J)**; Bonferroni correction was also used for **(C)**].

Mechanistic target of rapamycin complex 1 is activated by amino acids and by the phosphatidylinositol 3-kinase (PI3K)/Akt pathway, while it is inactivated by the AMPK (28, 29). ETEC infection inhibited the PI3K/Akt pathway and activated AMPK in piglet jejunum (Figures 4A,B). Thus, the activation of mTORC1 in response to ETEC infection may result from changes in amino acid concentration in the jejunum. Using GC–MS analysis, we found that eight amino acids significantly changed in abundance after ETEC infection (Figure 4C). The relative abundance of spermidine, glutamine, asparagine, citrulline, and ornithine decreased, while the relative abundance of glycine, taurine, and GABA increased after ETEC infection (Figure 4C). However, there was little change in mRNA expression of the activating transcription factor 4 (Figure 4D), an indicator of amino acid starvation (30), suggesting there was no overall amino acid starvation after ETEC infection.

As GABA was the most significantly increased amino acid (about fourfold), we hypothesized that GABA is responsible for increased IL-17 expression and mTORC1 activation during ETEC infection. Indeed, numerous investigations have shown that GABA signaling promotes mTORC1 activation (31–33). Treating mice with bicuculline (an antagonist of GABAA receptors) or with CGP-35348 (an antagonist of GABAB receptors) lowered *Il-17* expression in the jejunum during ETEC infection (Figure 4E). Bicuculline and CGP-35348 treatment also reversed the activation of mTORC1–EGR-2 signaling in mouse jejunum induced by ETEC infection (Figure 4F). GABA injection promoted *Il-17* expression in mouse jejunum during ETEC infection, while rapamycin prevented expression of *Il-17* caused by GABA injection (Figure 4G). Furthermore, treatment with l-allylglycine or semicarbazide (blockers of synthetic enzyme for GABA) reduced *Il-17* expression in mouse jejunum during ETEC infection (Figure 4H). l-Allylglycine or semicarbazide treatment also prevented the activation of mTORC1–EGR-2 signaling in mouse jejunum caused by ETEC infection (Figure 4I).

To investigate whether GABA signaling regulates Th17 cell differentiation, naïve CD4^+^ T cells were isolated and differentiated into Th17 cells under Th17 polarization conditions. In normal Th17 differentiation condition, about 3.0% of CD4^+^ T cells were differentiated into CD4^+^IL-17^+^ T cells after 3 days of differentiation; while l-allylglycine or semicarbazide decreased the percentage of CD4^+^IL-17^+^ T cells (Figure 4J). After 3 days of differentiation under normal Th17 polarization conditions, CD4^+^ T cells had higher abundance of phosphorylated mTORC1, compared to the naïve CD4^+^ T cells (Figure 4K). Also, l-allylglycine decreased the abundance of phosphorylated mTORC1 (Figure 4K). Collectively, GABA signaling promotes IL-17 expression during infection though mTORC1–EGR-2 signaling.

### Microbiota-Derived GABA Mediates IL-17 Expression during ETEC Infection

**Figure 5:**
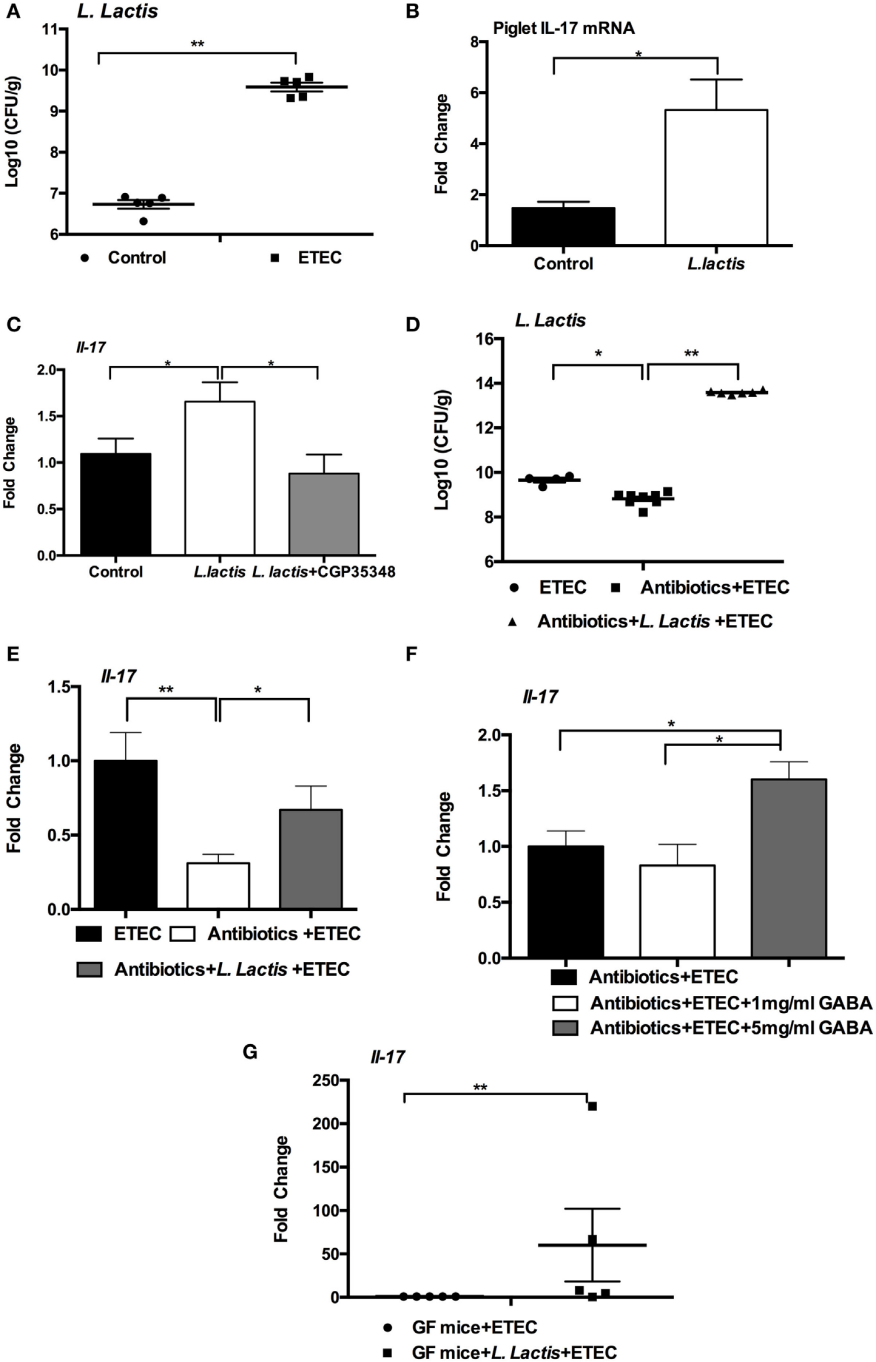
**Intestinal microbiota affects interleukin-17 (IL-17) expression**. **(A)** Bacterial counting of *Lactococcus lactis* subsp. *lactis* in enterotoxigenic *Escherichia coli* (ETEC)-infected mice and control mice. The jejunal contents were collect at 6-h post-ETEC infection for bacterial counting. **(B)** The mRNA expression of IL-17 in the jejunum was analyzed in piglets (*n* = 5). **(C)** The mRNA expression of IL-17 in the jejunum was analyzed in mice (*n* = 8). **(D)** Bacterial counting of *L. lactis* subsp. *lactis* in indicated models. **(E)** The mRNA expression of IL-17 in the jejunum was analyzed in indicated models. **(F)** The mRNA expression of IL-17 in the jejunum was analyzed in indicated models. **(G)** The mRNA expression of IL-17 in the mesenteric lymph node was analyzed in germ-free mice. Germ-free mice received *L. lactis* subsp. *lactis* or not at 1 week before ETEC infection. mRNA expression is relative to GF mice without *L. lactis* subsp. *lactis* administration (defined as 1; *n* = 5). Results shown are representative of two independent experiments with *n* = 5–8 **(A,D,E)** in each experiment, or of three independent experiments with *n* = 8 **(F)** in each experiment. *indicates a statistically significant difference between two groups (*p* < 0.05), while **indicates *p* < 0.01 [Mann–Whitney test **(A,G)**; unpaired *t* test with Welch’s correction **(B)**; one-way ANOVA **(C–F)**].

Intestinal microbiota has critical roles in GABA production from glutamate (34, 35) and in regulation of intestinal IL-17 expression (36, 37). Our previous study has found that ETEC-infected piglets had a higher percentage of *Lactococcus*, compared to the controls (manuscript submitted). This result was supported by our current finding that ETEC-infected mice had higher numbers of *L. lactis* subsp. *lactis*, as compared with uninfected controls (Figure 5A). Within the intestinal microbiota, *L. lactis* subsp. *lactis* is one of the main GABA-producing strains (38, 39). Thus we hypothesized that the increased abundance of intestinal *L. lactis* subsp. *lactis* during ETEC infection is responsible for activated GABA–IL-17 signaling in the context of ETEC infection. Indeed, we found that piglets orally inoculated with *L. lactis* subsp. *lactis* (ATCC19435) has higher mRNA expression of IL-17 in the jejunum compared to the piglets without *L. lactis* subsp. *lactis* inoculation (Figure 5B). The GABA production activity of *L. lactis* subsp. *lactis* (ATCC19435) is about 210.4 µM (38, 39). Similar to the observation from piglets, *L. lactis* subsp. *lactis* inoculation in mice significantly promoted the expression of *Il-17* in the jejunum compared to the mice without *L. lactis* subsp. *lactis* inoculation (Figure 5C). More importantly, inhibition of GABA signaling by CGP-35348 decreased the expression of *Il-17* in the jejunum of mice with *L. lactis* subsp. *lactis* inoculation (Figure 5C). To further test this hypothesis, we treated mice with a mixture of antibiotics for 1 week to decrease the load of *L. lactis* subsp. *lactis* before ETEC infection. To increase the abundance of *L. lactis* subsp. *lactis* in antibiotics-treated mice, we orally inoculated the *L. lactis* subsp. *lactis* to antibiotics-treated mice. The antibiotics-treated mice had a lower numbers of *L. lactis* subsp. *lactis* than the mice without antibiotics treatment (Figure 5D). After ETEC infection, we found that antibiotics-treated mice had lower expression of *Il-17* in the jejunum compared to the mice without antibiotics treatment (Figure 5E). Oral inoculation of *L. lactis* subsp. *lactis* before ETEC infection increased the numbers of *L. lactis* subsp. *lactis* in the content of jejunum and rescued the expression of *Il-17* during ETEC infection (Figures 5D,E). Interestingly, in antibiotics-treated mice, higher dosage of GABA supplementation also rescued the expression of *Il-17* in the jejunum during ETEC infection (Figure 5F). In germ-free mice, *L. lactis* subsp. *lactis* administration before ETEC infection also promoted expression of *Il-17* (Figure 5G). Collectively, intestinal GABA-producing bacteria have vital importance in IL-17 expression during ETEC infection.

### GABA–mTORC1 Signaling Mediates IL-17 Expression in Other Models

**Figure 6:**
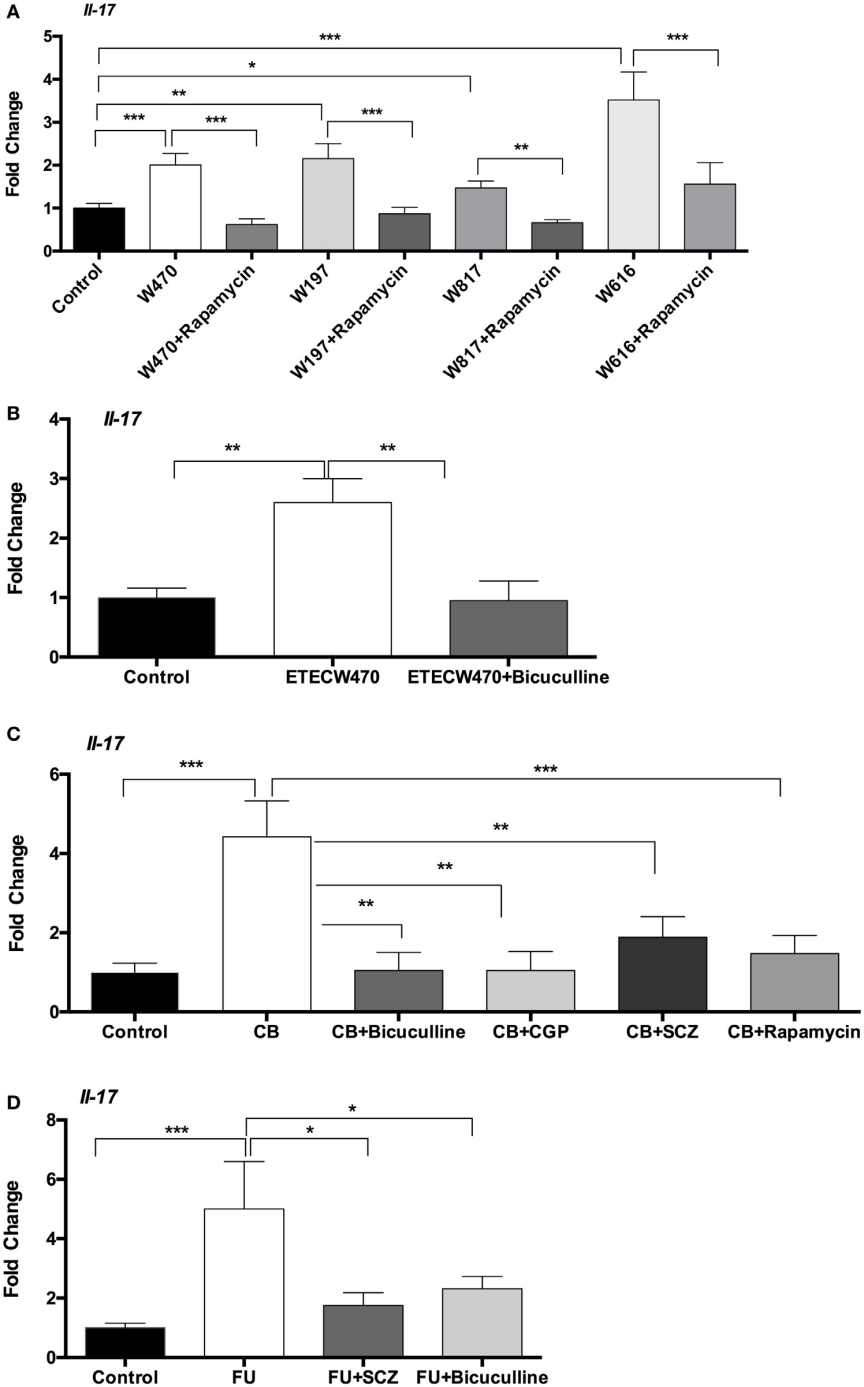
**γ-Aminobutyric acid (GABA)-mechanistic target of rapamycin complex 1 (mTORC1) signaling mediates mRNA expression interleukin-17 (IL-17) in other models**. **(A)** Rapamycin prevents mRNA expression of IL-17 induced by W470, W197, W817, or W616 (*n* = 10). **(B)** Bicuculline prevents mRNA expression of IL-17 induced by W470 (*n* = 10). **(C)** Inhibition of GABA–mTORC1 signaling inhibits mRNA expression of IL-17 in the colon during *Citrobacter rodentium* infection (*n* = 10). **(D)** Inhibition of GABA signaling inhibits the increase in mRNA expression of IL-17 induced by 5-fluorouracil (5-FU) (*n* = 10). *indicates a statistically significant difference between two groups (*p* < 0.05), while **indicates *p* < 0.01, and ***indicates *p* < 0.001 [one-way ANOVA **(A–D)**].

To validate the role of GABA–mTORC1 signaling in IL-17 expression in other intestinal infectious models, we infected mice with different ETEC strains and with Shiga-like toxin producing *E. coli*. In mouse model, *E. coli* W470, W197, W817, and W616 increased *Il-17* expression in the jejunum at 6-h postinfection (Figure 6A). Pretreatment with rapamycin prevented expression of *Il-17* in the jejunum caused by W470, W197, W817, and W616 at 6-h postinfection (Figure 6A). Similar to our previous W25K infection, bicuculline treatment reversed the increase in *Il-17* expression caused by W470 in mouse jejunum (Figure 6B). In *C. rodentium* infected mice, the expression of *Il-17* in the colon was higher than in control mice (Figure 6C). Interestingly, higher abundance of *L. lactis* subsp. *lactis* was also observed in the colon during *C. rodentium* infection (7.7 ± 0.03 log_10_ CFU/g in infected mice vs. 6.9 ± 0.08 log_10_ CFU/g in control mice). Rapamycin treatment lowered the colonic expression of *Il-17* (Figure 6C), indicating that inhibition of mTORC1 pathway reduced the expression of *Il-17* in the colon during *C. rodentium* infection. Furthermore, the inhibition of GABA signaling by semicarbazide, bicuculline, or CGP-35348 also lowered the expression of *Il-17* in the colon in the context of *C. rodentium* infection (Figure 6C). 5-Fluorouracil (5-FU) is frequently used in cancer treatment and also induces intestinal inflammation (40, 41). 5-FU enhanced *Il-17* expression in the jejunum at 6-h postinjection (Figure 6D). Semicarbazide or bicuculline treatment prevented the increase in *Il-17* expression caused by 5-FU (Figure 6D). In summary, GABA–mTORC1 signaling also functions in intestinal IL-17 expression in other intestinal inflammatory models.

## Discussion

Besides promoting local inflammation, emerging evidence shows that Th17 cells secrete IL-26, which kills extracellular bacteria through membrane-pore formation and triggers the production of interferon alpha through binding to bacterial DNA (42). Thus, Th17 response has a critical role in protection against extracellular bacterial infection. Similar to previous studies (15, 16), we observed that ETEC infection promotes intestinal IL-17 expression in piglet and mouse models. The increased expression of IL-17 during ETEC infection may come from adaptive cells (e.g., Th17 cells), or innate immune cells, such as innate lymphoid cells-3, γδ T cells, and NKT cells (11, 13). Unlike previous conclusion that increased IL-17 is mainly produced by γδ T cells at 5-h post-*E. coli* infection (25), this study indicates that a significant portion of the IL-17 comes from Th17 cells at 6-h post-ETEC infection. However, intestinal Th17 cell responses after bacterial infection (e.g., *C. rodentium* and *E. coli* O157) needs the epithelial adhesion of pathogens and is observed at 5–14 days postinfection (43, 44). Thus, the exact cellular source for increase IL-17 expression at 6-h post-ETEC infection needs further investigation. Indeed, enteric iTh17 has been observed at early time of C*itrobacter* and *Salmonella* infection (12).

Mechanistic target of rapamycin complex 1 is a conserved Ser/Thr protein kinase that functions as a sensor of growth factors, nutrients, and energy to promote anabolic cellular metabolism and functions as a master controller of cell growth (45). Recent studies have shown that mTORC1 signaling is crucially involved in Th17 cells differentiation and IL-17 expression (26). Inhibition of mTORC1 signaling with rapamycin reduces IL-17 expression in various inflammatory models (46–48). Similarly, we showed that mTORC1 signaling mediates the expression of IL-17 after intestinal ETEC infection, and this result is similar to the previous reports that mTORC1 signaling has a critical role in IL-17 expression (26). Likewise, another study has shown that rapamycin treatment inhibits the expression of IL-17 during *Campylobacter jejuni* infection (49). mTORC1–S6K1–EGR-2–GFI-1 axis is involved in IL-17 expression in ETEC infection. GFI-1, a downstream target of mTORC1–S6K1 axis (26), is a negative regulator of IL-17 expression and Th17 differentiation, and the downregulation of GFI-1 is important for IL-17 expression (26). However, the expression of IL-17 in the context of ETEC infection is independent of the S6K2 mediating nuclear translocation of RORγt, and HIF-1α pathway, which promotes IL-17 expression through: (a) transcriptional activation of RORγt in the cytoplasm, (b) collaboration with RORγt to promote IL-17 expression in the nucleus, through mechanisms involving p300 recruitment and histone acetylation, and (c) inhibition of Treg differentiation by targeting Foxp3 for ubiquitination and proteasomal degradation (26). Interestingly, ETEC infection reduces the abundance of HIF-1α. Although the underlying mechanisms are unknown, our results may indicate a complex regulatory role for mTORC1 in HIF-1α production, Th17 differentiation, and IL-17 expression.

We found that GABA signaling mediates intestinal IL-17 expression in ETEC infection through activation of mTORC1 signaling. In starvation conditions that normally inhibit mTORC1 signaling, the addition of GABA also increases S6 phosphorylation, suggesting that GABA signaling activates mTORC1 signaling even in starvation conditions (33). Recently, GABA has been suggested to exert a potential anti-inflammatory action by inhibiting major inflammatory events mediated by different immune cells. For example, increasing GABAergic activity ameliorates inflammation in experimental autoimmune encephalomyelitis (50). Oral GABA treatment ameliorates the inflammatory process both in non-obese diabetic mice (51) and in a mouse model of rheumatoid arthritis (52). Also, GABA_A_ agonists are beneficial in experimental encephalomyelitis (50) and allergic asthma (53), and GABA_B_ agonists exhibit a positive effect in dermatitis models (54). However, few investigations have addressed the possible action of GABA on intestinal inflammation during infection. Different from these previous reports (50–54), here we found that GABA promotes intestinal IL-17 expression. Similarly, dendritic cells increase GABA secretion *in vitro* after *Toxoplasma gondii* infection and exhibit a hyper-migratory phenotype because increased GABA activates GABAA receptor-mediated currents in *T. gondii*-infected dendritic cells (55). The inactivation of GABA signaling in *T. gondii*-infected dendritic cells by inhibition of GABA synthesis or transportation, or by blockade of GABAA receptor, results in impaired function of dendritic cells *in vitro*, including transmigration capacity, motility, and chemotactic response to CCL19 (55).

The gut microbiota affects numerous biological functions (56, 57) and is linked to the pathogenesis of various diseases, such as obesity (58), cancer (59), and liver cirrhosis (60). The influence of the gut microbiota on host physiological functions and the pathogenesis of disease may result from the activity of the microbiome and its metabolic products (56). Gut microbes produce various biologically active molecules, such as GABA, short-chain fatty acids, and biogenic amines (such as histamine), to actively regulate host health and disease states (56). For example, *Lactobacillus rhamnosus* JB-1 converts glutamate into GABA and alters GABA signaling in the brain, resulting in a reduction in stress-induced corticosterone, and in anxiety- and depression-related behavior (61). We found that ETEC infection induces a dysbiosis of the intestinal microbiota, especially promoting a higher percentage of GABA-producing *L. lactis* in the jejunum. Intestinal GABA-producing *L. lactis* has critical role in IL-17 expression during infection. However, the *L. lactis* used for GABA production in this study is not an inhabitant of the GI tract of either mice or piglets, thus it is interesting to test the function of GABA-producing *L. lactis* isolated from pigs. Also, it is interesting to explore the mechanism responsible for increased abundance of *L. lactis* during ETEC infection. Besides GABA, whether other products from *L. lactis*, or other members of intestinal microbiota are also involved in the pathogenesis of ETEC infection remain to know.

In conclusion, we propose the following scenario for how intestinal pathogens (i.e., ETEC) mediates intestinal IL-17 expression. During ETEC infection, ETEC induces the dysbiosis of gut microbiota, increasing GABA-producing *L. lactis* subsp. *lactis*. The increased content of GABA promotes IL-17 expression during infection through mTORC1–S6K1–EGR-2–GFI-1 pathway. This discovery has great potential to manipulate intestinal GABA–mTORC1 signaling and intestinal GABA-producing bacteria to treat intestinal chronic inflammatory diseases involving IL-17 expression. Our findings further support the notion that functional amino acids (e.g., glutamate and glutamine) play an important role in the immunity and health of animals (62–64).

## Author Contributions

WR, PH, TL, BT, and YY designed the experiments; WR, JY, HX, and SC conducted the experiments; GL, NL, and YP helped with animal experiments; WR and JY analyzed the data; ZY provided the TCR delta knockout mice; BZ, WL, and HW provided the germ-free mice; WR wrote the manuscript; GW, PH, and YY revised the manuscript.

## Conflict of Interest Statement

The authors declare that the research was conducted in the absence of any commercial or financial relationships that could be construed as a potential conflict of interest.

## Abbreviations

EGR-2, early growth response protein 2; ETEC, enterotoxigenic *Escherichia coli*; Foxp3, forkhead box P3; GABA, γ-aminobutyric acid; GFI-1, growth factor independent 1; HIF-1α, hypoxia-inducible factor 1 α; IL-17, interleukin-17; mTORC1, mechanistic target of rapamycin complex 1; PCNA, proliferating cell nuclear antigen; RORγt, RAR-related orphan receptor gamma t; S6K, ribosomal protein S6 kinase.
