# Supplementary material for: Intestinal Microbiota-Derived GABA Mediates Interleukin-17 Expression during Enterotoxigenic Escherichia coli Infection
Source: Front Immunol. 2017 Jan 16;7:685. doi: 10.3389/fimmu.2016.00685 (PMC5237640; doi:10.3389/fimmu.2016.00685)
Supplement: Supplementary file 1 [file Table_1.docx]

**Table S1**. Primers used in this study.

| Primer | Sequence (5’-3’) |
| --- | --- |
| Mouse b-actin-forward | CCTGTATGCCTCTGGTCGTA |
| Mouse b-actin-reverse | CCATCTCCTGCTCGAAGTCT |
| Mouse IL-17-forward | TACCTCAACCGTTCCACGTC |
| Mouse IL-17-reverse | TTTCCCTCCGCATTGACAC |
| Mouse-Foxp3-forward | CCCAGGAAAGACAGCAACCTT |
| Mouse-Foxp3-reverse | TTCTCACAACCAGGCCACTTG |
| Mouse-Hif-a-forward | AGCTTCTGTTATGAGGCTCACC |
| Mouse-Hif-a-reverse | TGACTTGATGTTCATCGTCCTC |
| Mouse-Rorc-forward | CCGCTGAGAGGGCTTCAC |
| Mouse-Rorc-reverse | TGCAGGAGTAGGCCACATTACA |
| Mouse-Gif1-forward | AGGAACGCAGCTTTGACTGT |
| Mouse-Gif1-reverse | GATGAGCTTTGCACACTGGA |
| Mouse-Egr2-forward | CTACCCGGTGGAAGACCTC |
| Mouse-Egr2-reverse | AATGTTGATCATGCCATCTCC |
| Pig-b-actin-forward | CTGCGGCATCCACGAAACT |
| Pig-b-actin-reverse | AGGGCCGTGATCTCCTTCTG |
| Pig IL-6-forward | CAAAGCCACCACCCCTAAC |
| Pig IL-6-reverse | TCGTTCTGTGACTGCAGCTT |
| Pig IL-8-forward | TTCTTCTTTATCCCCAAACTGG |
| Pig IL-8-reverse | CCACATGTCCTCAAGGTAGGA |
| Pig IL-17-forward | CTCTCGTGAAGGCGGGAATC |
| Pig IL-17-reverse | GTAATCTGAGGGCCGTCTGG |
| Pig TNF-a-forward | TTCCTCACTCACACCATCAGCC |
| Pig TNF-a-reverse | TGCCCAGATTCAGCAAAGTCC |
